# Efficacy of Cyclooxygenase-2 Inhibitors for Headache in Acute Brain Injury: A Systematic Review

**DOI:** 10.21203/rs.3.rs-4232407/v1

**Published:** 2024-04-08

**Authors:** Hector David Meza Comparan, Anum Khaliq, Luciola Martins Frota, Daniela Pomar-Forero, Bakhtawar Ahmad, Erica Marnet, Fernanda J.P. Teixeira, Anita Thomas, Priyank Patel, Haley Brunkal, Saanvi Singireddy, Brandon Lucke-Wold, Carolina B. Maciel, Katharina M. Busl

**Affiliations:** Department of Neurology, Division of Neurocritical Care, University of Florida, College of Medicine, Gainesville, FL 32611, USA; Department of Neurology, Division of Neurocritical Care, University of Florida, College of Medicine, Gainesville, FL 32611, USA; Department of Neurology, Division of Neurocritical Care, University of Florida, College of Medicine, Gainesville, FL 32611, USA; Department of Neurology, Division of Neurocritical Care, University of Florida, College of Medicine, Gainesville, FL 32611, USA; Department of Neurology, Division of Neurocritical Care, University of Florida, College of Medicine, Gainesville, FL 32611, USA; Department of Medicine, Bridgeport Hospital, Bridgeport, CT, USA 06610; Department of Neurology, University of Miami/Jackson Memorial Hospital, FL, USA, 33130; Department of Neurology, Division of Neurocritical Care, University of Florida, College of Medicine, Gainesville, FL 32611, USA; Department of Neurology, Division of Neurocritical Care, University of Florida, College of Medicine, Gainesville, FL 32611, USA; Department of Neurology, Division of Neurocritical Care, University of Florida, College of Medicine, Gainesville, FL 32611, USA; Department of Neurology, Division of Neurocritical Care, University of Florida, College of Medicine, Gainesville, FL 32611, USA; Department of Neurosurgery, University of Florida, College of Medicine, Gainesville, FL 32611, USA; Department of Neurology, Division of Neurocritical Care, University of Florida, College of Medicine, Gainesville, FL 32611, USA; Department of Neurosurgery, University of Florida, College of Medicine, Gainesville, FL 32611, USA; Department of Neurology, Yale University School of Medicine, New Haven, CT 06520, USA; Department of Neurology, University of Utah, Salt Lake City, UT 84132, USA; Department of Neurology, Division of Neurocritical Care, University of Florida, College of Medicine, Gainesville, FL 32611, USA; Department of Neurosurgery, University of Florida, College of Medicine, Gainesville, FL 32611, USA

**Keywords:** Acute Brain Injury, Analgesics, Cyclooxygenase 2 Inhibitors, Headache, Pain Management, Systematic Review

## Abstract

**Background:**

Headache management after acute brain injury (ABI) is challenging. While opioids are commonly used, selective cyclooxygenase-2 inhibitors (COXIBs) may be promising alternatives. However, concerns about cardiovascular effects and bleeding risk have limited their use. We aimed at summarizing available data on efficacy of COXIBs for headache management following ABI.

**Methods:**

A systematic review was conducted through MEDLINE and Embase for articles published through 09/2023 (PROSPERO CRD42022320453). No language filters were applied to the initial searches. Interventional or observational studies and systematic reviews assessing efficacy of COXIBs for headache in adults with ABI were eligible. Article selection was performed by two independent reviewers using Distiller SR^®^. Descriptive statistics were used for data analysis, while meta-analysis was unfeasible due to study heterogeneity.

**Results:**

Of 3190 articles identified, six studies met inclusion criteria: four randomized controlled trials and two retrospective cohort studies, all conducted in neurosurgical patients (total n=738) between 2006–2022. Five studies used COXIBs in the intervention group only. Of the six studies, four found a reduction in overall pain scores in the intervention group, while one showed improvement only at 6 hours postoperative, and one did not find significant differences. Pain scores decreased between 4–15%, the largest shift being from moderate to mild severity. Three studies found an overall opioid use reduction throughout hospitalization in the intervention group, while one reported a reduction at 12 hours postoperative only. Opioid consumption decreased between 9–90%. Two studies found a decrease in hospital-length-of-stay by ~1 day in the intervention group. The one study reporting postoperative hemorrhage found a statistically non-significant 3% reduction in the intervention group.

**Conclusions:**

In adults with ABI, COXIBs may serve as opioid-sparing adjunctive analgesics for headache control, with limited but pointed data to indicate efficacy in the post-neurosurgical setting. However, further safety data remains to be elucidated.

## Introduction

The wide spectrum of acute brain injury (ABI) featuring headaches includes subarachnoid hemorrhage (SAH), traumatic brain injury (TBI), ischemic and hemorrhagic strokes, neurosurgical interventions requiring craniotomy or craniectomy, and intracranial infections [1]. The overall estimated incidence of headaches in patients with ABI ranges from 25 to 78% [2–4]. Up to 40% of patients admitted to a neurointensive care unit report headaches [5]. The pathophysiology of headaches following ABI involves multiple mechanisms including cell membrane dysfunction, axonal injury, disruption of the blood-rotransmitter and hormone release, as well as the onset of an inflammatory cascade [6]. The multifaceted pathophysiology and different types of ABI with different headache manifestations render headache management following ABI a complex issue. Furthermore, available treatment options are limited, and pain control is often suboptimal [1].

Frequently used medications include acetaminophen, opioids, non-steroidal anti-inflammatory drugs (NSAIDs), corticosteroids, and antiseizure medications [1].

Opioids remain the core of management for severe headaches for many patients with ABI [7–10], despite the potential negative impact of opioids on neurologic examination [11] and recovery [12]. Alarmingly, patients with ABI may be uniquely susceptible to opioid misuse [13,14], emphasizing the need for analgesic alternatives.

Cyclooxygenase-2 (COX-2) is an inflammatory mediator that is overexpressed immediately following ABI. It leads to the production of reactive oxygen species and prostaglandin metabolites and exacerbates the initial insult [15,16]. Selective COX-2 inhibitors (COXIBs) have been shown to decrease progressive inflammation, edema, and secondary injury in preclinical ABI models [17] and are hypothesized to improve outcomes in patients with ABI [15]. While the anti-inflammatory role of COXIBs in ABI has been explored, their role in headache control in this population is less well characterized. Despite their promising potential for headache management following ABI, literature has primarily focused on the utilization of COXIBs in patients with rheumatologic diseases [18], cancer [19], and orthopedic surgery [20,21], to address both systemic inflammation and analgesia. COXIBs have led to decreased morphine requirements for patients who underwent shoulder and knee arthroplasties, as well as laparoscopic cholecystectomies [20–22]. Hence, it is conceivable that COXIBs could present an analgesic alternative for patients with ABI.

We aimed to assess the data on the efficacy of COXIBs for headache management in adult patients with ABI through a systematic review of the available literature. We hypothesized COXIBs would reduce headache intensity in patients with ABI with an opioid-sparing effect.

## Methods

### Protocol and Registration

This systematic review was reported in accordance with the Preferred Reporting Items for Systematic Reviews and Meta-Analyses (PRISMA) statement recommendations [23]. The protocol was registered *a priori* on PROSPERO (CRD42022320453).

### Data Sources and Search Strategy

An electronic database search was conducted in MEDLINE and Embase for articles published from inception through September 2023. The original literature searches were conducted between August 2021 and February 15^th^, 2022, and an updated search was run through September 2023. The search strategy was developed by the research team in consultation with a Health Librarian and included a combination of keywords related to the concepts of ABI, headache, and COXIBs. No language, study design, or year of publication filters were applied to the initial searches. The complete search strategy is outlined in **Supplementary File 1**.

### Eligibility criteria

Articles eligible for inclusion met the following criteria: 1) Interventional (randomized controlled trials —RCT —, non-RCT, or adaptive clinical trials) or observational human subject studies (cross-sectional, case-control, prospective or retrospective cohort studies, case reports or case series), or systematic reviews (with or without meta-analysis), 2) Studies conducted in adult patients (defined as 17 years of age or older) who had experienced an ABI resulting in headache, 3) Studies assessing headache management with COXIBs in at least one of the study arms, regardless of comparator(s) used, and 4) Availability of full-text publication in English (original or translated). Preclinical studies, correspondence papers, expert opinions, and editorials were excluded.

### Outcomes

Outcomes of interest included pain scale scores (regardless of the scale used), opioid use at any timepoint(s) during hospitalization, hospital-length-of-stay (LOS), and any adverse events reported.

### Study selection

All articles identified by the literature searches were exported into DistillerSR^®^ for screening, following deduplication using EndNote^™^. During level 1 screening, two independent reviewers screened titles and abstracts according to eligibility criteria in a blinded process, and any conflicts were resolved by a third reviewer. All relevant studies identified after level 1 underwent full-text review for eligibility during level 2 screening. Eligible articles after full-text assessment were included for data extraction. The reference lists of studies that underwent data extraction were scrutinized for any additional suitable articles.

### Data extraction and synthesis

Data were extracted from included articles using a tailored data extraction sheet, which included the year of publication, study design, definition of intervention and control groups, sample size, and study outcomes. Due to the large methodological heterogeneity observed, a meta-analysis was not deemed feasible. Instead, a narrative synthesis of the data with a tabulation of study characteristics was created.

### Assessment of the risk of bias

Two independent reviewers assessed the risk of bias for each one of the included studies using the revised Cochrane risk-of-bias tool for randomized trials (RoB 2) [24] and the Newcastle-Ottawa Quality Assessment Scale adapted for cohort studies [25], for interventional and observational studies, respectively. The review team resolved any conflicts using discussion until a consensus was reached. For the purposes of the review, based on an approach previously used [26], a score lower or equal to 5 on the Newcastle-Ottawa Quality Assessment Scale was classified as low quality, 6 to 7 as medium quality, and greater or equal to 8 as high quality. Of note, utilizing these tools instead of the Quality in Prognosis Studies (QUIPS) tool for the risk of bias assessments constitutes an amendment from our originally registered protocol; the tools applied were deemed the appropriate tools for the studies included in our final review.

## Results

**Figure 1:**
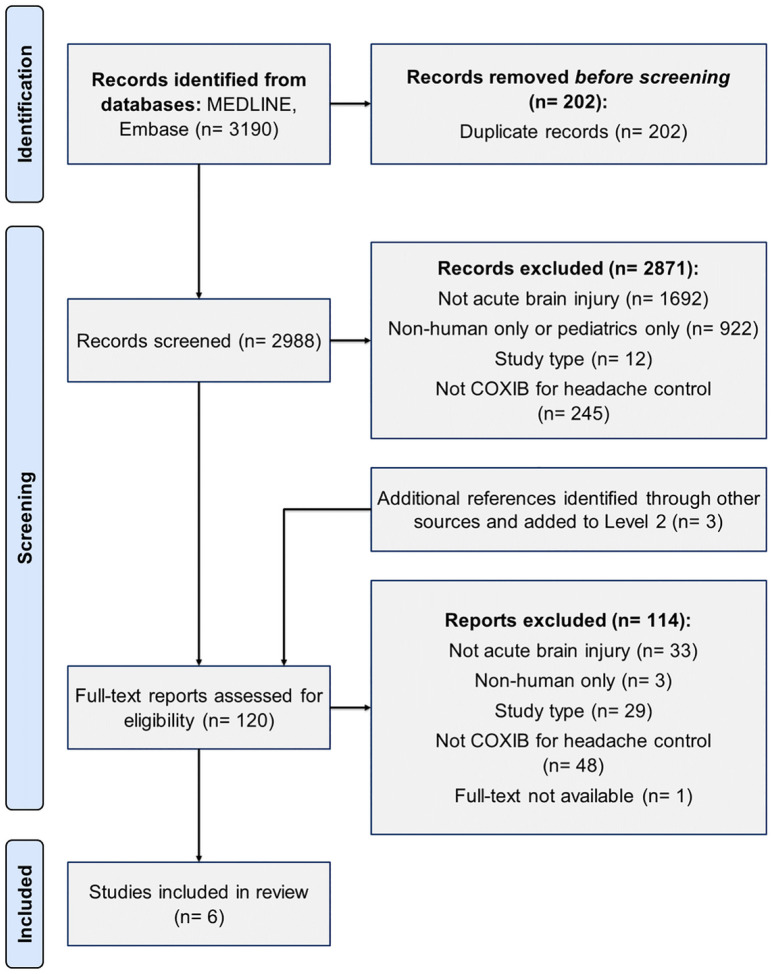
PRISMA flow diagram

The initial search yielded a total of 3190 articles. Following the removal of duplicates, 2988 articles were screened, with 6 studies meeting eligibility criteria (Figure 1): four RCT [27–30] and two retrospective cohort studies [31,32]. All studies were conducted in neurosurgical patients (n=738), between 2006–2022. No systematic reviews or meta-analyses pertinent to our objective were retrieved.

Individual study characteristics of the six included studies are summarized in Table 1. Five studies assessed the efficacy of COXIBs for headache control in the intervention versus control groups [27,28,30–32], and one study assessed two different pain regimens employing COXIBs, in both the intervention and control groups [29]. The specific COXIBs used, dosing, frequency, timing, and routes of administration varied; parecoxib 40 mg IV was most frequently employed (n=264) [27,29–31], followed by celecoxib (n=93) [32] and rofecoxib (n=14) [28]. Pain scores were reported as mean or median at different time points, from the immediate postoperative period (0 hours) up to 72 hours. Opioid use was reported as mean dose, median tablets, or mean morphine equivalent units (MEU), and hospital LOS was reported as mean or median days. Adverse events reported included incidence of postoperative nausea and vomiting, and postoperative hemorrhage.

### Pain scores

In all studies, pain scores ranged between mild and moderate severity. Four studies noted decreased pain scores at overall time points for the intervention groups compared with control groups [28,29,31,32], while one study only did so at one point in time (6 hours postoperative) [27]. Overall, pain scores dropped between 4–15%, with the largest shift observed from moderate to mild severity.

One study found no statistically significant differences in pain scores between the study arms [30].

### Opioid use

Three studies found a decrease in opioid use throughout the hospital course for the intervention groups [28,29,32], whereas one study noted a reduction only at 12 hours postoperative [27]. Overall, opioid consumption decreased by 9–90%.

One study found no differences in opioid use among the groups [30], while one did not report this outcome [31].

### Hospital LOS

Two studies showed a reduction in hospital LOS for the intervention groups by approximately 1 day [28,29], while two others showed no statistically significant differences between groups [31,32], and the remaining two did not report on this outcome [27,30].

### Adverse events

Four studies reported any adverse events [29–32], with one reporting a statistically significant lower incidence of postoperative nausea and vomiting in the intervention group [29]. Only one study —a retrospective cohort assessing the efficacy and safety of an opioid-sparing protocol (which included celecoxib) compared to an opioid-based regimen, in patients undergoing cranial surgery— reported on the incidence of postoperative hemorrhage, which found a statistically non-significant 3% reduction in the intervention group (5% vs 8%, p=0.527) [32].

### Assessment of the risk of bias

**Figure 2:**
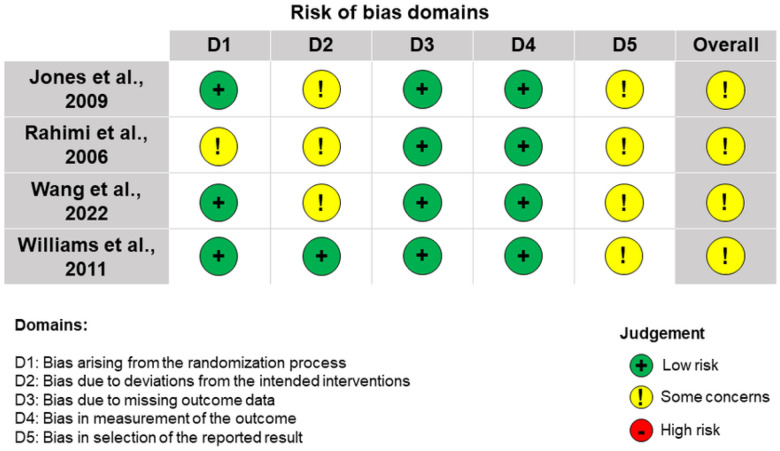
Risk of bias assessments using the Cochrane RoB 2 tool for randomized parallel-group trials Rahimi et al. [28] were judged as having “some concerns” for D1 due to a lack of clarity in allocation concealment. Jones, Rahimi, and Wang [27–29] were deemed as having “some concerns” in D2 due to missing intention-to-treat analyses. All studies were judged as having “some concerns” in D5 due to a lack of pre-specified statistical analysis plans or references to trial protocols.

Risk of bias assessments for the RCT included in our review are presented in Figure 2. “Some concerns” were present for bias arising from the randomization process, bias due to deviations from the intended interventions, and bias in the selection of the reported result.

Risk of bias assessments for the observational studies included in our review are presented in Table 2. Neither study was given any points for the “representativeness of the exposed cohort” item, and one study was assigned no points for the Comparability category [31].

## Discussion

Our systematic review yielded 6 studies pertinent to the use of COXIBs for headache in ABI of one subtype—post-neurosurgical patients. In this subpopulation of patients, utilization of COXIBs for analgesia resulted in a statistically significant pain reduction in two-thirds of the studies and decreased opioid use in half of the studies, suggesting a role for COXIBs in this setting. However, their safety profile in this population remains largely unknown, as only one study reported on postoperative hemorrhage —which found a 3% reduction in the intervention group, though lacked statistical significance.

To our knowledge, this is the first systematic review of the literature exclusively addressing the efficacy of COXIBs for headache management in ABI. A previous systematic review by Galvin et al. evaluated pharmacological interventions for the prevention of postoperative headache in patients undergoing craniotomy [33], and included studies evaluating several classes of NSAIDs (COXIBs, diclofenac, dexketoprofen, dipyrone, and ibuprofen), as well as dexmedetomidine, gabapentin, pregabalin, acetaminophen, scalp infiltrations, and scalp blocks. In this review, the authors found a reduction in pain up to 24 hours postoperative when using NSAIDs, compared to control or placebo. At the time of their review, not all the data we included were available. Our results include newer studies on the topic of COXIBs [29,31,32] and more pointedly show the distinct impact of COXIBs—showing reductions in overall pain scores with the use of COXIBs [28,29,31,32]— and as such provide a novel addition to the slim, cumulative body of knowledge on headache management in the neurocritically ill. Of note, one study in our review that did not find differences in pain scores when COXIBs were used was conducted exclusively in patients undergoing supratentorial craniotomy [30]; it is possible that the selection of the examined population in this study contributed to the lack of difference: severe pain is highest with infratentorial craniotomy [1,34].

While the prior review by Galvin et al. [33] found no clear benefit for NSAIDs in reducing opioid requirements, we found a reduction of opioid use in three of the included studies (all using multimodal analgesia approaches) [28,29,32], emphasizing the potential for COXIBs within multimodal pain regimens.

Cumulative data further contrasts with the findings of data from an RCT by Ryan et al. [35], in which patients undergoing elective craniotomy received either rofecoxib 50 mg (n=19) or placebo (n=15), 1 hour before surgery, without reported differences between intervention and control groups for pain scores, opioid use, or incidence of adverse events—however, the results of this trial are only reported as abstract form and hence could not be included in our systematic review. Limiting factors in this trial from the information available in the abstract are a small sample size, a single-dose oral regimen, and potentially, the timing of administration.

Outside of the neurosurgical setting, the efficacy of COXIBs for headache management in ABI has not been well investigated. A retrospective cohort study [15] conducted on the premise of anti-inflammatory effects compared the outcomes of patients with TBI treated with celecoxib versus a matched control group and found a higher 1-year survival and lower medical complication rates. However, pain-related outcomes were not reported. Moreover, Begemann et al. [36] conducted a systematic review and meta-analysis of RCT testing anti-inflammatory medications in patients with TBI; this review did not yield studies utilizing COXIBs, reflecting further the current gap in the literature.

The gap of data on COXIBs may partially be rooted in concerns about increased risk of cardiovascular events rapidly hampering clinical use after the initial market release [32], although specifically rofecoxib (which is no longer in the market), but not celecoxib, was responsible for this increased cardiovascular risk [37]. In fact, celecoxib has been associated with a lower risk of stroke or cardiovascular death compared to other NSAIDs such as diclofenac or ibuprofen [38,39]. Additional advantages of COXIBs include avoidance of the gastrointestinal adverse effects (e.g., gastric ulcers, bleeding, and perforation) typically associated with non-selective COX inhibitors [40], and lack of antiplatelet qualities (as opposed to other NSAIDs) which, altogether, make the use of COXIBs ideal for headache management following ABI, especially when the expected risk of bleeding is high [27,41,42].

### Limitations

Several limitations need to be considered when interpreting our findings. First, the study population included in our review is primarily comprised of craniotomy patients; hence, our findings are not generalizable to other patients with ABI. Second, the heterogeneity in study designs, pain assessments, intervention with different routes and dosing of COXIBs, and mostly small sample sizes precluded the calculation of pooled estimates for outcomes of interest.

Third, the level of details available regarding outcomes assessment was underreported in the study by Rahimi et al. [28], which did not specify the associated time points. Fourth, it is difficult to estimate the precise effect of COXIBs on pain reduction and adverse events in the studies by Wang et al. [29] and Ahmad et al. [32], which evaluated COXIBs as part of a multimodal analgesia approach and used preoperative patient counseling. Fifth, in the two studies where there was minimal or no impact on pain scores or opioid use with COXIBs [27,30], the intervention was only applied once intraoperatively, possibly rendering it insufficient to observe any statistically significant differences among the study groups. Sixth, although utilization of COXIBs for analgesia resulted in a statistically significant pain reduction in two-thirds of the included studies, observed improvements in pain scores could arguably be deemed as clinically relevant, as the largest shift achieved was by 1.5 points. Lastly, the adverse events reported in our included studies solely consisted of postoperative nausea and vomiting and postoperative hemorrhage, with only one study showing a statistically significant difference in the incidence of adverse events between the intervention and control groups [29], specifically postoperative nausea and vomiting, preventing us from drawing any firm conclusions on the safety profile of COXIBs in our population.

## Conclusions

In our review, we found moderate-quality evidence that, in patients undergoing neurosurgery, COXIBs reduce headache intensity in the postoperative and might decrease opioid requirements, especially in the context of craniotomy. Future research efforts should be aimed at designing and conducting prospective studies —preferably RCT— testing the safety and efficacy of COXIBs for headache management in other forms of ABI, including SAH, intracerebral hemorrhage, TBI, ischemic stroke, and neuroinfectious diseases, with careful adjudication of adverse events while minimizing the effect of potential confounders such as concomitant administration of other analgesics.

## Figures and Tables

**Table 1:** Summary of individual study characteristics

study	Year of publication	study design	Intervention	Control	Sample size and population	Results: Intervention vs control	Summary
Rahimi et al.	2006	RCT	Rofecoxib 25 mg twice daily PO*	Placebo*	n = 27 Adults undergoing elective craniotomy	10-point VAS^a^: 3.8 ± 0.9 vs 5.3 ± 1.3, p = 0.003 Percocet intake^b^: 4.5 [0–14] tablets vs 14 [3–34] tablets, p = 0.011 Morphine use^b^: 6 [0–18] mg vs 10 [0–40] mg, p = 0.073 Hospital LOS^a^: 3 ± 0.7 days vs 4.2 ± 1.8 days, p = 0.044	Intervention provides better pain control, decreases opioid use (Percocet), and reduces LOS
Wang et al.	2022	RCT	Perioperative multimodal analgesia (intraoperative scalp blocks with 0.5% ropivacaine, dexmedetomidine and parecoxib 40 mg IV)	Routine analgesia**	n = 151 Adults undergoing elective supra- and infratentorial craniotomy	10-point NRS^a^: 2.5 ± 0.8/2.0 ± 1.0/1.5 ± 1.2/0.9 ± 1.0/0.7 ± 0.8/0.4 ± 0.7 vs 2.9 ± 0.8/2.9 ± 1.2/2.6 ± 1.3/2.2 ± 1.1/1.7 ± 0.9/1.7 ± 1.5, p < 0.005 Intraoperative remifentanil use^c^: 0.7 [0.6, 1.0] mg vs 0.9 [0.7, 1.4] mg, p < 0.0001 Hospital LOS^c^: 3 [2, 4] days vs 4 [4, 6] days, p < 0.0001 Adverse events (PONV)^d^: 7 [9.2%] vs 21 [28.0%], p = 0.003	Intervention provides better pain control, decreases opioid use, and reduces LOS and incidence of adverse events
Zhu et al.	2022	Retrospective cohort study	Parecoxib 40 mg IV***	Placebo (saline)***	n = 200 Adults undergoing glioma resection (awake craniotomy)	10-point NRS^e^: 2.5/2.0/1.8 vs 3.0/2.5/2.0, p < 0.05 Hospital LOS^a^: 11.4 ± 4.1 vs 10.3 ± 3.2, p = 0.89 Adverse events (PONV)^d^: 6 [6%] vs 5 [5%], p > 0.05	Intervention may provide better pain control, as demonstrated in three out of five time points (2,12 and 24 hours postoperative), with no effect on LOS or adverse events
Ahmad et al.	2021	Retrospective cohort study	Stepwise opioid-sparing protocol (celecoxib PQ, acetaminophen and ketorolac)	Stepwise opioid-based analgesia protocol (acetaminophen, hydrocodone and IV morphine)	n = 184 Adults undergoing cranial surgery	10-point DVPRS^e^: 3.45/3.21/2.90 vs 4.19/4.00/3.59, p < 0.05 MEU^e^: 1.2/0.8/1.6/4.6 vs 11.6/13.0/12.6/45.2, p < 0.001 Hospital LOS^e^: 1.85 days vs 2.24 days, p = 0.184 Adverse events (postoperative hemorrhage)^d^: 5 [5%] vs 7 [8%], p = 0.527	Intervention may serve as an opioid-sparing alternative for pain control, with no effect on LOS or adverse events
Jones et al.	2009	RCT	Parecoxib 40 mg IV	Placebo (saline)	n = 80 Adults undergoing elective craniotomy	100-point VAS^f^: 25 [3] vs 38 [4], p = 0.01 Morphine use^f^: 0.8 [0.3] mg vs 2.7 [0.8] mg, p = 0.03	Intervention only reduced pain scores and opioid use at one out of four time points (at 6 hours and 6–12 hours postoperative, respectively)
Williams et al.	2011	RCT	Parecoxib 40 mg IV	Placebo (saline)	n = 96 Adults undergoing supratentorial craniotomy	10-point VRS^g^: Dynamic pain: 6.0/4.0/3.0/3.0/2.0/2.0 vs 4.0/3.0/5.0/4.0/4.0/3.0, p >0.05 Resting pain: 5.0/4.0/4.0/2.0/2.0/1.0 vs 2.0/3.0/3.0/3.0/2.0/2.0, p >0.05 Morphine use^b^: 16 [1–92] mg vs 20 [0–102] mg, p = 0.38 Adverse events (PONV)^d^: 24 [51%] vs 28 [56%], p = 0.55	Intervention did not reduce pain scores, opioid use or incidence of adverse events

Data are presented as ^a^mean±SD, ^b^median [range], ^c^median [IQR], ^d^frequency [percentage], ^e^mean, ^f^mean [SEM], and ^g^median

* In addition to Percocet (oxycodone/acetaminophen 5/325 mg) and morphine

** Per the individual practice of the anesthesiologist, usually COXIBs

*** In addition to dexmedetomidine and remifentanil

Abbreviations: DVPRS: defense and veterans pain rating scale; IQR: interquartile range; IV: intravenous; LOS: length of stay; NRS: numerical rating scale; PO: per os (by mouth); PONV: postoperative nausea and vomiting; RCT: randomized controlled trial; SD: standard deviation; SEM: standardized error of the mean; VAS: visual analog scale; VRS: verbal rating scale

**Table 2:** Risk of bias assessments using the Newcastle-Ottawa Quality Assessment Scale adapted for cohort studies

Study	Selection				Comparability	Outcome			Quality score (out of 9)
	Representativeness of the exposed cohort	Selection of the nonexposed cohort	Ascertainment of exposure	Demonstration that outcome of interest was not present at start of study	Comparability of cohorts on the basis of the design or analysis	Assessment of outcome	Sufficient follow-up	Adequacy of follow-up	
Ahmad et al., 2021	-	*	*	*	**	*	*	*	8
Zhu et al., 2022	-	*	*	*	-	*	*	*	6

The populations in both studies were not considered representative of the exposed cohorts, since all subjects were identified from single institutions. Zhu et al. [31] were assigned no points for the Comparability category considering it did not control for any variables, as opposed to Ahmad et al. [32] which used propensity score matching to reduce baseline differences between the groups
